# Ophthalmic infections in children presenting to Angkor Hospital for Children, Siem Reap, Cambodia

**DOI:** 10.1186/1756-0500-7-784

**Published:** 2014-11-05

**Authors:** Phara Khauv, Paul Turner, Channy Soeng, Sona Soeng, Catrin E Moore, Rachel Bousfield, Nicole Stoesser, Kate Emary, Duy Pham Thanh, Stephen Baker, Vu thi Ty Hang, H Rogier van Doorn, Nicholas PJ Day, Christopher M Parry

**Affiliations:** Angkor Hospital for Children, Siem Reap, Cambodia; Cambodia-Oxford Medical Research Unit, Angkor Hospital for Children, Siem Reap, Cambodia; Mahidol-Oxford Tropical Medicine Research Unit, Faculty of Tropical Medicine, Mahidol University, Bangkok, Thailand; Centre for Tropical Medicine, Nuffield Department of Medicine, University of Oxford, Oxford, UK; The Hospital for Tropical Diseases, Wellcome Trust Major Overseas Programme, Oxford University Clinical Research Unit, Ho Chi Minh City, Vietnam; The London School of Hygiene and Tropical Medicine, London, United Kingdom

**Keywords:** Paediatric, Ophthalmic, Infection, Chlamydia

## Abstract

**Background:**

Ophthalmic infections cause significant morbidity in Cambodian children but aetiologic data are scarce. We investigated the causes of acute eye infections in 54 children presenting to the ophthalmology clinic at Angkor Hospital for Children, Siem Reap between March and October 2012.

**Findings:**

The median age at presentation was 3.6 years (range 6 days – 16.0 years). Forty two patients (77.8%) were classified as having an external eye infection, ten (18.5%) as ophthalmia neonatorum, and two (3.7%) as intra-ocular infection. Organisms were identified in all ophthalmia neonatorum patients and 85.7% of patients with an external eye infection. Pathogens were not detected in either of the intra-ocular infection patients. Most commonly isolated bacteria were *Staphylococcus aureus* (23 isolates), coagulase-negative staphylococci (13), coliforms (7), *Haemophilus influenzae*/*parainfluenzae* (6), *Streptococcus pneumoniae* (4), and *Neisseria gonorrhoeae* (2). *Chlamydia trachomatis* DNA was detected in 60% of swabs taken from ophthalmia neonatorum cases.

**Conclusions:**

This small study demonstrates the wide range of pathogens associated with common eye infections in Cambodian children. The inclusion of molecular assays improved the spectrum of detectable pathogens, most notably in neonates.

## Findings

Ophthalmic infections are a major cause of acute and chronic morbidity in Cambodian children, and one of the commonest causes of visual impairment in this population [1–3]. However, there are limited data on the spectrum of microbial organisms causing these infections in Cambodia, since diagnostic laboratory facilities and ophthalmology services are scare, with an estimate of less than twenty trained ophthalmologists practising in the entire country (http://www.hollows.org.au/our-work/where-we-work-country/cambodia). The aim of this study was to make a preliminary assessment of the causes of infections in children presenting to the ophthalmic service at Angkor Hospital for Children (AHC) in Siem Reap to aid empirical prescribing practice, inform public health interventions and establish a surveillance baseline for the monitoring of disease trends. The study protocol was reviewed and approved by the Institutional Review Board at Angkor Hospital for Children, Cambodia and the Oxford Tropical Ethics Committee, UK (ref 46-11).

Between 1^st^ March and 31^st^ October 2012, children presenting to the AHC eye clinic with clinical evidence of ophthalmic infection were invited to participate in the study and written consent obtained from the attending parent or caregiver. Swabs, corneal scrapings, or intra-ocular specimens were submitted for microscopy and bacterial culture, according to the clinical diagnosis. Specimens were transported to the onsite microbiology immediately after collection and were cultured on a variety of culture media (all specimens: 5% sheep blood agar, chocolate agar, MacConkey agar, Sabouraud agar, fastidious anaerobe agar (Oxoid, Basingstoke, UK; prepared in house); if a specimen from a neonate: additional selective plate for isolation of *Neisseria gonorrhoeae*; if corneal scrape or intra-ocular pus specimen: additional culture in thioglycollate broth with subculture to fastidious anaerobe agar after 48 hours) and incubated for up to 48 hours aerobically, anaerobically, or in 5-10% CO_2_ as appropriate. Cultured organisms were identified by colony morphology, Gram stain characteristics, and standard microbiologic techniques [4]. Antimicrobial susceptibilities were performed by disk diffusion according to current Clinical Laboratory Standards Institute (CLSI) guidelines [5]. Benzyl penicillin and ceftriaxone minimum inhibitory concentrations (MIC) were determined by Etest (bioMerieux, Marcy L’Etoile, France) for *Streptococcus pneumoniae* isolates with a 1 μg oxacillin disc zone diameter of <20 mm, and interpreted using the CLSI guidelines. In addition to bacterial culture, an aliquot of each specimen was stored at -80°C in 200 μL sterile phosphate buffered saline (PBS) for molecular analysis. Nucleic acids were extracted from the stored specimen aliquots using the NucliSENS easyMag system (bioMerieux, Marcy L’Etoile, France) and *Chlamydia trachomatis*, adenovirus, and herpes simplex virus (HSV) 1 and 2 infections were detected by real-time PCR assays, as previously described [6–8].

Fifty four patients were enrolled in the study. The median age at presentation was 3.6 years (interquartile range 0.3 – 11.1 years; range 6 days – 16.0 years). Thirty patients (30/54; 55.6%) were male. Forty two patients (42/54; 77.8%) were classified as having an external eye infection (conjunctivitis (11), corneal ulcer (11), dacrocystitis (7), stye (7), eyelid abscess (6)), ten (18.5%) as ophthalmia neonatorum, and two (3.7%) as intra-ocular infection. At least one bacterial or viral species was detected from specimens submitted for 46 patients (46/54; 85.2%; Table 1): more than one organism was detected in a third of specimens (17/54; 31.5%; Table 2). Potentially pathogenic organisms were identified in all (10/10) ophthalmia neonatorum patients and 85.7% (36/42) of patients with an external eye infection. There was no growth from specimens in both of the intra-ocular infection patients. Antimicrobial susceptibility test results for commonly cultured organisms are shown in Table 3. *Chlamydia trachomatis* DNA was detected in swabs from seven patients (7/54; 13.0%): six were ophthalmia neonatorum presentations and one of these was a co-infection with *Neisseria gonorrhoeae*. HSV DNA was detected from a swab in one child presenting with corneal ulceration. Adenovirus DNA was not detected in any specimens.

**Table 1:** **Culture and PCR results by category of infection**

Organism	Intra-ocular infection	External eye infection	Ophthalmia neonatorum	Total
	N(column%)	N(column%)	N(column%)	N(column%)
**Number** **(** **row** **%)**	2 (3.7)	42 (77.8)	10 (18.5)	54
**Culture-** **based detection**				
*Staphylococcus aureus*	0 (0)	19 (45.2)	4 (40.0)	23 (42.6)
CoNS^1^	0 (0)	9 (21.4)	4 (40.0)	13 (24.1)
Gram positive bacilli	0 (0)	3 (7.1)	4 (40.0)	7 (13.0)
Enterobacteriaceae^2^	0 (0)	4 (9.5)	3 (30.0)	5 (9.3)^3^
*Haemophilus influenzae*	0 (0)	4 (9.5)	0 (0)	4 (7.4)
*Streptococcus pneumoniae*	0 (0)	4 (9.5)	0 (0)	4 (7.4)
*Haemophilus parainfluenzae*	0 (0)	1 (2.4)	1 (10.0)	2 (3.7)
*Neisseria gonorrhoeae*	0 (0)	0 (0)	2 (20.0)	2 (3.7)
*Pseudomonas aeruginosa*	0 (0)	2 (4.8)	0 (0)	2 (3.7)
*Burkholderia pseudomallei*	0 (0)	1 (2.4)	0 (0)	1 (1.9)
*Candida* sp.	0 (0)	1 (2.4)	0 (0)	1 (1.9)
*Enterococcus* sp.	0 (0)	0 (0)	1 (10.0)	1 (1.9)
*Moraxella catarrhalis*	0 (0)	0 (0)	1 (10.0)	1 (1.9)
*Streptococcus bovis*	0 (0)	0 (0)	1 (10.0)	1 (1.9)
**PCR-** **based detection**				
Adenoviruses (AdV)	0 (0)	0 (0)	0 (0)	0 (0)
Herpes Simplex viruses (HSV)	0 (0)	1 (2.4)	0 (0)	1 (1.9)
*Chlamydia trachomatis*	0 (0)	1 (2.4)	6 (60.0)	6 (11.1)

^1^Coagulase-negative staphylococci.

^2^Coliforms: *Klebsiella pneumoniae* (2), *Enterobacter cloacae* (1), *Escherichia coli* (1), *Morganella morganii* (1), *Pantoea* sp. (1), *Serratia plymuthica* (1).

^3^Number (%) of specimens from which at least one member of the Enterobacteriaceae was isolated (there were two swabs with two species of Enterobacteriaceae isolated).

**Table 2:** **Culture details of the seventeen polymicrobial infections**

Diagnosis	Gram positive	Gram negative	Other organisms
Conjunctivitis	*Staphylococcus aureus*	*Haemophilus influenzae*	*Chlamydia trachomatis*
	*Streptococcus pneumoniae*		
Conjunctivitis	CoNS^1^	*Pantoea* sp.	
		*Serratia plymuthica*	
Conjunctivitis	*S. aureus*	*Pseudomonas aeruginosa*	
Conjunctivitis	*S. aureus*	*H. parainfluenzae*	
Conjunctivitis	CoNS	*H. influenzae*	
Conjunctivitis	CoNS	*H. influenzae*	
	Gram positive bacillus		
Conjunctivitis	*S. pneumoniae*		
	CoNS		
Corneal ulcer		*Enterobacter cloacae*	
		*Morganella morganii*	
Dacrocystitis	CoNS		
	Gram positive bacillus		
Stye	*S. aureus*		*Candida* sp.
Ophthalmia neonatorum	*S. aureus*	*Neisseria gonorrhoeae*	*C. trachomatis*
Ophthalmia neonatorum	*S. aureus*	*Moraxella catarrhalis*	
Ophthalmia neonatorum	*Enterococcus sp*.	*H. parainfluenzae*	
	*S. aureus*	*Klebsiella pneumoniae*	
Ophthalmia neonatorum	*S. aureus*	*K. pneumoniae*	*C. trachomatis*
	CoNS		
Ophthalmia neonatorum	*Streptococcus bovis*		*C. trachomatis*
	Gram positive bacillus		
Ophthalmia neonatorum	CoNS	*N. gonorrhoeae*	
Ophthalmia neonatorum	CoNS		*C. trachomatis*
	Gram positive bacillus		

^1^Coagulase-negative staphylococci.

**Table 3:** **Antimicrobial susceptibility results of commonly cultured organisms**

Organism	Drug(%susceptible)							
	AMP ^1^	AMC ^1^	OX ^1^	P ^1^	CRO ^1^	CIP ^1^	SXT ^1^	CN ^1^
*S. aureus*	ND^2^	ND	95.7	4.4	ND	100	95.7	100
*S. pneumoniae*	ND	ND	ND	100	100	ND	25.0	ND
*H. influenzae*	83.3	100	ND	ND	100	100	40.0	ND
*N. gonorrhoeae*	ND	ND	ND	0	100	0	ND	ND
Enterobacteriaceae	42.9	85.7	ND	ND	83.3	85.7	85.7	85.7

^1^AMP – ampicillin; AMC – co-amoxiclav; OX – oxacillin; P – penicillin; CRO – ceftriaxone; CIP – ciprofloxacin; SXT – co-trimoxazole; CN - gentamicin.

^2^Not done/not applicable.

This study, although small, demonstrates the wide range of pathogens responsible for common eye infections in Cambodian children. These organisms are generally similar to those found in reports from other populations [9, 10]. Variations between study results may reflect age distribution of cases, contact lens use (a risk factor for keratitis), and host factors such as malnutrition and immunisation status [11–13]. However, the absence of confirmed adenovirus or fungal infection was surprising and perhaps attributable to the small sample size, the limited duration of the study (which have resulted in a missed seasonal adenovirus outbreak), specimen factors (i.e. most specimens were swabs), and the short incubation period for fungal culture plates (although fungal elements were not seen on microscopy in any of the specimens). Larger studies in this population maybe warranted to validate the current findings. The isolation of *Burkholderia pseudomallei*, the cause of melioidosis, from an eyelid abscess is noteworthy and demonstrates the diversity of presentation of infections with this organism [14]. *Staphylococcus aureus* was the most frequently cultured organism and the majority (22/23; 95.7%) of isolates were meticillin sensitive, although with the emergence of community-acquired meticillin-resistant *S. aureus* (MRSA) infections in Cambodia this may change with time [15]. Isolates of *Neisseria gonorrhoeae* were penicillin and fluoroquinolone resistant but susceptible to ceftriaxone, which is similar to the findings of other eye infection studies from Southeast Asia [16, 17]. The addition of molecular testing increased the spectrum of detectable pathogens, most notably in neonates where 60% of infections were associated with detection of *Chlamydia trachomatis* DNA. Maternal screening for sexually transmitted infections (STI) in pregnancy and tetracycline eye ointment administration for newborns are not routine practice in Cambodia but may be desirable given the frequent isolation of STI-related organisms from ophthalmia neonatorum cases. These data will serve as a useful baseline for further studies of these important infections in Cambodia.
